# Zinc Oxide Nanoparticles for Water Purification

**DOI:** 10.3390/ma14164747

**Published:** 2021-08-23

**Authors:** Angela Spoială, Cornelia-Ioana Ilie, Roxana-Doina Trușcă, Ovidiu-Cristian Oprea, Vasile-Adrian Surdu, Bogdan Ștefan Vasile, Anton Ficai, Denisa Ficai, Ecaterina Andronescu, Lia-Mara Dițu

**Affiliations:** 1 Department of Science and Engineering of Oxide Materials and Nanomaterials, Faculty of Applied Chemistry and Materials Science, University Politehnica of Bucharest, 1-7 Gh Polizu Street, 011061 Bucharest, Romania; angela.8317@gmail.com (A.S.); cornelia_ioana.ilie@upb.ro (C.-I.I.); adrian.surdu@upb.ro (V.-A.S.); bogdan.vasile@upb.ro (B.Ș.V.); ecaterina.andronescu@upb.ro (E.A.); 2 National Centre for Micro and Nanomaterials and National Centre for Food Safety, Faculty of Applied Chemistry and Materials Science, University Politehnica of Bucharest, Spl. Indendentei 313, 060042 Bucharest, Romania; truscaroxana@yahoo.com (R.-D.T.); ovidiu73@yahoo.com (O.-C.O.); denisaficai@yahoo.ro (D.F.); 3 Department of Inorganic Chemistry, Physical Chemistry and Electrochemistry, Faculty of Applied Chemistry and Materials Science, University Politehnica of Bucharest, 1-7 Gh Polizu Street, 050054 Bucharest, Romania; 4 Academy of Romanian Scientists, 3 Ilfov Street, 050045 Bucharest, Romania; 5 Faculty of Biology, University of Bucharest, 1-3 Aleea Portocalelor, 060101 Bucharest, Romania; lia_mara_d@yahoo.com

**Keywords:** ZnO, photocatalyst, antibacterial activity, water purification

## Abstract

In this study, zinc oxide nanoparticles were synthesized through a simple co-precipitation method starting from zinc acetate dihydrate and sodium hydroxide as reactants. The as-obtained ZnO nanoparticles were morphologically and structurally characterized by Fourier transform infrared spectroscopy (FTIR), X-ray diffraction (XRD), scanning electron microscopy (SEM), transmission electron microscopy (TEM), photocatalytic activity, and by determining the antimicrobial activity against Gram-negative and Gram-positive bacteria. The XRD pattern of the zinc oxide nanoparticles showed the wurtzite hexagonal structure, and its purity highlighted that the crystallinity correlated with the presence of a single product, zinc oxide. The ZnO nanoparticles have an average crystallite size of 19 ± 11 nm, which is in accordance with the microscopic data. ZnO nanoparticles were tested against methyl orange, used as a model pollutant, and it was found that they exhibit strong photocatalytic activity against this dye. The antibacterial activity of ZnO nanoparticles was tested against Gram-negative and Gram-positive strains (*Escherichia coli*, *Staphylococcus aureus*, and *Candida albicans*). The strongest activity was found against Gram-positive bacteria (*S. aureus*).

## 1. Introduction

Water is the most valued and important resource in the world and the lack of it has become a serious problem. Industrialization, urbanization, and climate change have created an urgent demand for the clean water that is so essential to human health. Lack of water can cause diseases such as typhoid fever, dysentery, cholera, and diarrhea, resulting in many deaths worldwide. Countless freshwater sources in the world are contaminated because of industrial activities and human negligence and they need to be purified [1,2].

Water purification involves removing undesirable chemicals, biological contaminants, or even suspended solids from water systems to produce clean, safe water for human consumption and other purposes. There are traditional methods that include processes such as filtration, sedimentation, distillation, and chlorination, but these have limitations and can be resistant to antibiotics. Researchers are trying to overcome these limitations by developing alternative methods [3].

Of all the diverse materials used in different purification methods, the nanomaterial that has attracted the most attention as an antibacterial agent is zinc oxide. The literature provides a multitude of synthesis methods for zinc oxide, such as thermal decomposition [4], spray pyrolysis [5], solvothermal reaction [6], forced hydrolysis [7], the sol-gel method or CVD [8], the hydrothermal method [9], and co-precipitation [10]. In addition, all the synthesis methods for ZnO presented above, microwave synthesis is considered a green chemistry approach, having processing advantages and being environmentally friendly [11,12]. ZnO is a multifunctional material with many areas of application such as optical devices [13], auto-cleaning paints [14], sunscreens [15], cosmetics [16], and photocatalysts [17,18]. Moreover, zinc oxide has numerous applications in diverse fields, such as drug delivery [19,20], cosmetics [16,21], paints [5], medical devices [22], dentistry [23], and the textile industry [24]. The applicability of zinc oxide is not limited to sunscreens [15]; it is also important in other industries such as rubber, pharmaceuticals [25], and food [26].

On the nanoscale, zinc oxide shows antimicrobial properties, which makes it a potential agent for various applications [27]. Researchers have successfully incorporated ZnO as an antimicrobial agent into textiles [28], surface coatings [29], cosmetics [16,21], and cellulose fibers [30] to inhibit microbial growth. For this reason, zinc oxide is widely accepted as a beneficial antibacterial agent and due to its durability and selectivity, is considered a safe material for humans and animals [31,32]. The U.S. FDA classifies ZnO as Generally Recognized as Safe (GRAS). Many studies have demonstrated that ZnO antibacterial activity has potential applications in water purification by improving the quality of wastewater [3,33]. The literature describes the photocatalytic mechanism of ZnO that influences its antibacterial activity and how light enhances it [22]. The safety and photocatalytic activity of ZnO have become important factors when referring to its antimicrobial activity and antimicrobial potential for water treatment [8,27,34,35,36].

The aim of this study was to use ZnO nanoparticles synthesized by precipitation in water treatment. ZnO nanoparticles were synthesized and characterized by the proper techniques and further evaluated for their antimicrobial and photodegradative potential. A photodegradation test on a pollutant model (organic dye) was conducted and the proven antimicrobial activity revealed that these nanoparticles are suitable for the desired applications.

## 2. Materials and Methods

### 2.1. Experimental

Zinc acetate dihydrate, Zn(OAc)_2_·2H_2_O, having 99.99% purity, was acquired from Sigma Aldrich (Merck, Burlington, MA, USA). Sodium hydroxide (NaOH) with 98% purity was obtained from Fluka (Merck, Burlington, MA, USA), and distilled water was used in this experiment. All chemicals used in the present study were of analytical grade without further purification.

For the experiments, we used the following laboratory devices: a hotplate magnetic stirrer from Daihan LabTech (Batam, Indonesia), model LMS-1003; a furnace from Nabertherm GmbH (Lilienthal, Germany), model L 9/11/B180; and an electric oven from Memmert GmbH (Büchenbach, Germany), type UF55.

We synthesized ZnO through a simplified co-precipitation method as described in [10]. To obtain zinc oxide nanoparticles, two solutions were prepared from zinc acetate dihydrate [Zn(OAc)_2_·2H_2_O], and sodium hydroxide [NaOH]. Both were dissolved in 50 mL of distilled water each until complete dissolution. The obtained solutions were combined and magnetically stirred at 1300 rpm on a hot plate at almost 70 °C until white precipitates of zinc oxide nanoparticles formed. After the precipitate was washed several times for acetate removal, it was left to dry in an electric oven at 60 °C overnight. The powders of zinc oxide were then placed in a furnace at 1000 °C for 3 h.

### 2.2. Characterization

The obtained ZnO nanoparticles were characterized by thermal analysis, Fourier transform infrared spectroscopy (FTIR), X-ray diffraction (XRD), scanning electron microscopy (SEM), transmission electron microscopy (TEM), photocatalytic activity, and by determining the antimicrobial activity against Gram-negative and Gram-positive bacteria.

The Fourier transform infrared spectroscopy (FTIR) measurements were performed using a Nicolet iS50R spectrometer (Thermo Fisher Scientific, MA, USA). The spectra were performed at room temperature using the attenuated total reflection (ATR) (Thermo Fisher Scientific, MA, USA), with 32 sample scans between 4000 and 400 cm^−1^ at a resolution of 4 cm^−1^, with the scanning time being 47 s. The recording and the future processing and analysis of the data were possible by connecting the spectrometer to the data acquisition and processing unit through the Omnic program (Thermo Fisher Scientific, MA, USA).

X-ray diffraction experiments were carried out on a Panalytical Empyrean instrument (Malvern Panalytical, Worcestershire, UK) with Ni-filtered Cu radiation (λ = 0.15406 Å) equipped with a 1/4° fixed divergence slit and a 1/2° anti-scatter slit on the incidence beam side, and a 1/2° anti-scatter slit mounted on a PIXCel3D detector (Malvern Panalytical, Worcestershire, UK) on the diffracted beam side. Data reduction and analysis of the patterns were performed in HighScore Plus 3.0.e software coupled with the ICDD PDF4 + 2021 database. For the Rietveld refinement [37] procedure, based on Hill and Howard’s computer program [38], a polynomial function and a pseudo-Voigt function were used for the approximation of the background and the peak profile, respectively. For the correction of intensity caused by preferential orientation, the March–Dollase [39] procedure was used.

The electron microscopy images were obtained using a Quanta Inspect F50 (FEI Company, Eindhoven, The Netherlands) equipped with a field emission gun (FEG) with a 1.2 nm resolution, and an energy dispersive X-ray spectrometer (EDS) with a MnK resolution of 133 eV Kα.

The transmission electron images were obtained on dried, finely powdered samples using a high-resolution Tecnai G2 F30 S-TWIN (FEI Company, Eindhoven, Netherlands) transmission electron microscope equipped with a selected area electron diffraction (SAED) (Gatan, Inc., Pleasanton, CA, USA) module. The microscope operated in bright-field transmission mode at an acceleration voltage of 300 kV, with a punctual and line resolution of 2 Å and 1 Å, respectively.

Thermal analysis (TG-DSC) was performed with an STA 449 F3 Jupiter apparatus, from Netzsch (Selb, Germany). Approximately 10 mg of dry powder was placed in an open alumina crucible and heated up to 900 °C with a 10 K∙min^−1^ rate, under a flow of 50 mL∙min^−1^ of dried air. As a reference, an empty alumina crucible was used. The evolved gases were analyzed with an FTIR Tensor 27 from Bruker (Bruker Co., Ettlingen, Germany) equipped with a thermostatic gas cell.

The photocatalytic activity was determined against methyl orange (MO) 6.11 × 10^−5^ M (20 mg/L) solution by irradiation with a LOHUIS^®^(Lohuis, Bucharest, Romania) a fluorescent lamp of 160 W/2900 lm, with a color temperature of 3200 K and a color rendering index of >60, placed at a 20 cm distance. A sample of 0.0250 g ZnO powder was placed in a 10 mL solution of MO and left under stirring for 30 min in the dark to reach adsorption–desorption equilibrium. After irradiation, at defined time intervals, a sample was placed in a quartz 10 mm cuvette, and its UV–Vis spectra were recorded with a JASCO (Easton, PA, USA) V560 spectrophotometer, with a speed of 200 nm∙min^−1^.

For the qualitative antimicrobial assay, we used the adapted diffusiometric method was (according to the CLSI recommendation, 2015). On the surface of the Mueller Hinton medium (without glucose) with 2% agar (pH = 7.2–7.4), Sabouraud medium agar with chloramphenicol, with a thickness of 4 mm, distributed in Petri dishes (Ø = 10 cm), was inseminated on the “canvas”, a standardized inoculum (suspension in a physiological buffer) obtained from the fresh culture (18–20 h) of *S. aureus* and *E. coli*, with a standard density of 1.5 × 10^8^ CFU/mL (corresponding with 0.5 McFarland standard), and *C. albicans* with a density of 3 × 10^8^ CFU/mL (corresponding to 1 McFarland standard).

The fresh cultures were obtained by inseminating the strains into the solid media trypticase soy agar (TSA) and yeast pepton glucose (YPG). Subsequently, 10 μL of each suspension of tested nanoparticles was observed on the surface of media inseminated in the canvas. After their diffusion, the Petri dishes were incubated for 18–24 h at 37 °C. The sensitivity of microbial strains to the action of ZnO nanoparticles was assessed using the diameters of the inhibition zones around the spot [26].

The quantitative assay was performed using the micro dilutions method in 96-well plates in liquid TSA medium or YPG to determine the minimum inhibitory concentrations (MICs). In this sense, binary serial dilutions were performed from each suspension of the tested nanoparticles in volumes of 150 μL of liquid media. Each well was inoculated with 15 μL of microbial suspension, with a standard McFarland density of 0.5 (for bacterial strains) and 1 (for yeast strains), and then incubated at 37 °C for 24 h. Each test was performed in a microbial culture control (wells containing only culture medium inoculated with microbial suspension) and with a media sterility control (wells containing only culture medium). After incubating the plates at 37 °C for 24 h, the samples were analyzed under macroscopic observation. The concentration of the tested compound corresponding to the last well in which the cultural development is no longer observed represents the MIC value (µg/mL). In subsequent wells, including cell growth control (C^+^) wells, media are disturbed because of microbial growth. The sterility control (C^−^) well does not show developed cultures [40].

The quantitative assay—investigating the capacity to inhibit adhesion to an inert substrate by the crystal violet method—was conducted to establish the effect of the tested suspensions on the adhesion capacity of the inert substrate before continuing to the MIC method. Adhesion to the inert substrate of the tested strains was evaluated at 24 h and 48 h after incubation by washing the wells 3 times with a sterile physiological buffer and fixing them with cold methanol for 5 min. After removing the methanol, the dry plates were stained with 1% crystal violet solution for 20 min. After staining, the excess dye was rinsed with water. The dye from the cells adhered to the walls and solubilized with 33% acetic acid solution. A spectrophotometric reading of the absorbance of the suspensions at 490 nm was taken, with the BioTek Synergy™ HTX ELISA Multi-Mode Reader (BioTek, Winooski, VT, USA) [26].

Antimicrobial assessments were performed in triplicate and were analyzed using GraphPad Prism 9 for Windows 64-bit, version 9.1.1 (225), developed by GraphPad Software, San Diego, CA, USA. We compared the resulting data using analysis of variance (ANOVA), and Tukey’s multiple comparisons test where a *p*-value < 0.05 was considered statistically significant.

## 3. Results and Discussion

**Figure 1:**
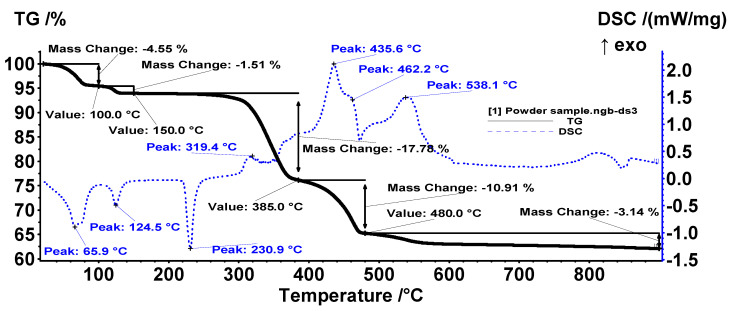
Thermal analysis of ZnO powder.

The ZnO nanoparticles were analyzed by TG/DSC to establish the sample purity and the calcination temperature (if necessary). The as-obtained ZnO precipitate still contained adsorbed water molecules, –OH moieties, and some zinc acetate species (Figure 1).

**Figure 2:**
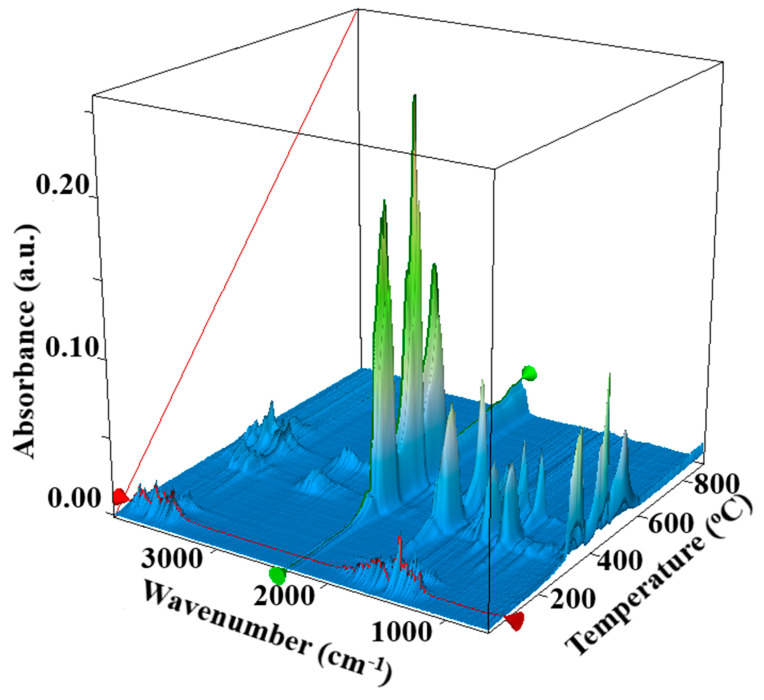
FTIR 3D chromatogram of evolved gases.

The first two mass loss processes correspond to the elimination of bound water molecules and –OH moieties from the nanoparticle’s surface. Endothermic effects accompany both mass loss steps. The FTIR 3D chromatogram confirms the presence of water molecules in the evolved gases up to 135 °C (Figure 2). The endothermic effect from 230.9 °C, without recorded mass loss, corresponds to the melting of Zn(CH_3_COO)_2_ impurities.

After 270 °C, the impurities start to decompose, eliminating acetate ions and, at the same time, an exothermic oxidation reaction to CO_2_ and H_2_O occurs. The FTIR spectra of evolved gases present the characteristic vibration for CH_3_COOH (3550 cm^−1^ ν_OH_, 2970 cm^−1^ ν_CH_, 1745 cm^−1^ δ_OH_) and CO_2_ (2324–2355 cm^−1^). The multistep mass loss process after 270 °C indicates the presence of more than one type of impurity, most probably Zn_5_(OH)_8_(CH_3_COO)_2,_ because it is also present in the sample. Based on the 2D representation, carbonate release is intense between 400 and 600 °C, but acetate release also appears in the same interval. As such, we decided to calcinate the sample at 1000 °C to remove all the impurities. This thermal treatment is also useful because the precursor is transformed into pure ZnO.

**Figure 3:**
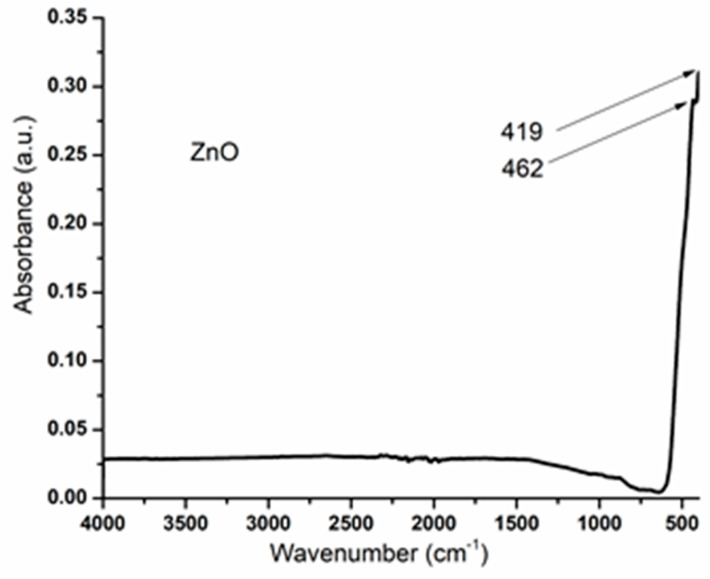
FTIR spectra of ZnO nanoparticles.

FTIR analysis was performed to evaluate the purity of the ZnO powder. The FTIR spectra of the synthesized ZnO samples was measured between 4000 and 400 cm^−1^, as presented in Figure 3. The main adsorption peaks are observed at 462 cm^−1^ and 419 cm^−1^ and correspond to the stretching vibrations of the Zn–O bond [41,42]. No absorption peaks are observed for the precursors (especially acetate). On the FTIR spectrum presented below, the main characteristic peaks can be identified as belonging to ZnO, and because no peaks belonging to acetate are present, we can conclude that pure ZnO powder was obtained [43].

**Figure 4:**
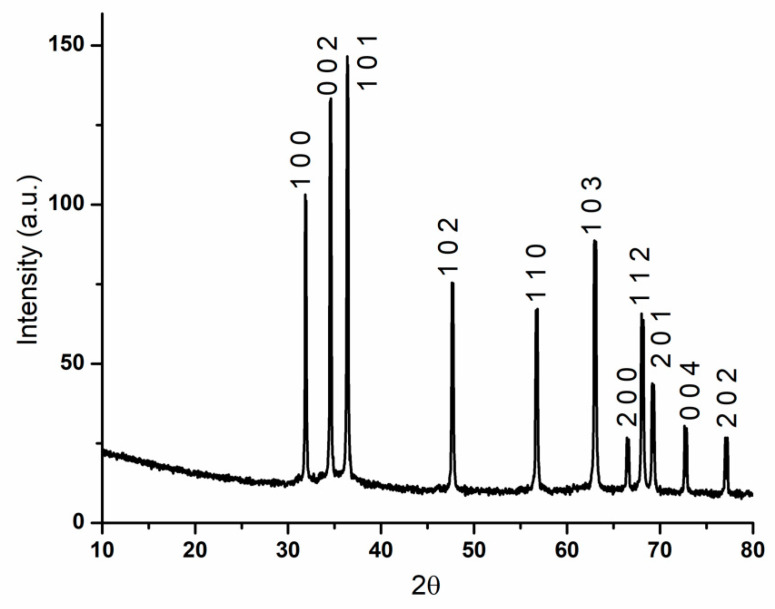
XRD pattern for ZnO nanoparticles.

The XRD pattern (Figure 4) of the synthesized ZnO nanoparticles shows the formation of a single-phase component, zinc oxide. The X-ray diffraction technique was used to determine the crystallinity degree of the prepared sample. The data in the pattern presented below were reduced and refined by the Rietveld algorithm. The agreement indices (R_expected_ = 9.1688, R_profile_ = 8.8184, weighted R_profile_ = 10.6262, and χ^2^ = 1.3432) show that the pattern fits with the ZnO hexagonal wurtzite structure (ICDD PDF4 + 04-007-1614 [44]), with lattice parameters of a = b = 3.251648 ± 0.000022 Å and c = 5.207833 ± 0.000041 Å. It is worth mentioning that the bond angles are α = β = 90, and γ = 120, and the unit cell volume is approximately 47.68644 Å^3^. Moreover, there is a preferential orientation in the 002 crystallographic direction with a March–Dollase parameter of 0.81842. The average crystallite size determined from the whole pattern fitting is 19 ± 11 nm.

**Figure 5:**
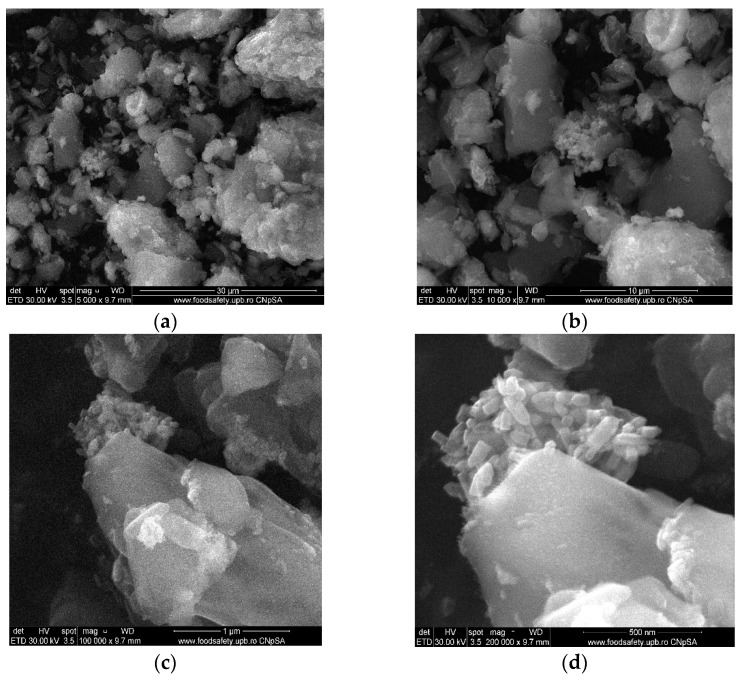
SEM images for ZnO powder, (**a**) 5000× magnification, (**b**) 10,000× magnification, (**c**) 100,000× magnification, (**d**) 200,000× magnification.

The SEM images (Figure 5) represent the as-obtained ZnO nanoparticles gathered in micrometric agglomerations arranged in a polyhedral-shaped manner. ZnO nanoparticles present uniformity in size and shape and the majority of them are hexagonal.

**Figure 6:**
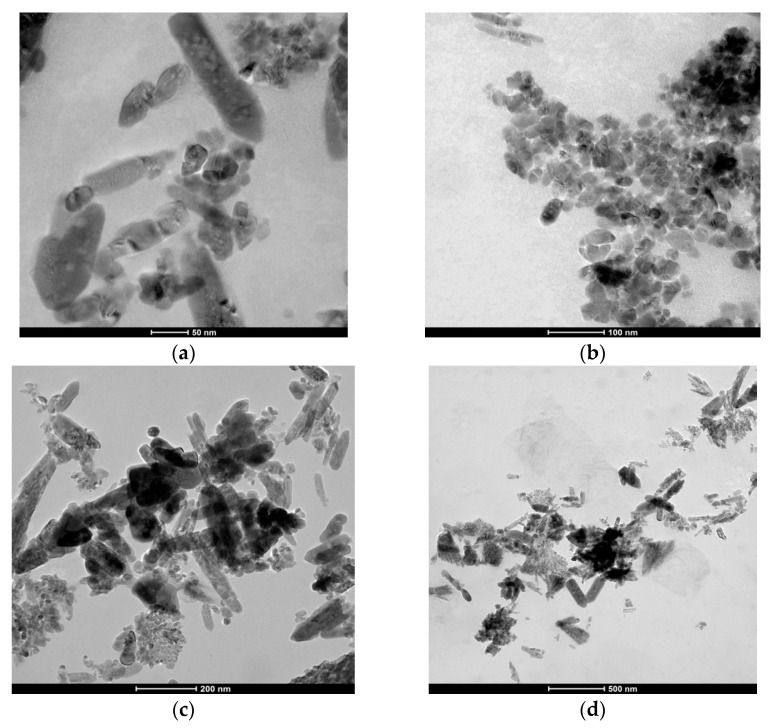
TEM images for ZnO nanoparticles, (**a**) scale bar 50 nm, (**b**) scale bar 100 nm, (**c**) scale bar 200 nm, (**d**) scale bar 500 nm.

The TEM images (Figure 6) obtained of ZnO nanoparticles reveal that the powder is composed of polyhedral-shaped particles, having a particle size ranging from 20 to 30 nm. By correlating TEM with XRD analysis, we conclude that the particles are mainly composed from one crystallite. Moreover, the nanopowder presents a tendency to form small agglomerates with discontinuous and irregular grading.

**Figure 7:**
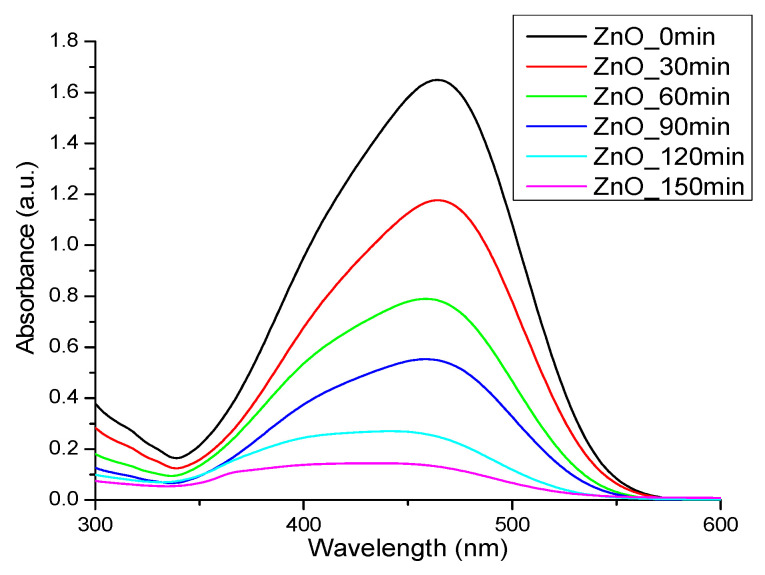
Photocatalytic activity against methyl orange (MO) for ZnO nanoparticles.

The photocatalytic activity of ZnO nanoparticles is well-known, and the literature reports many investigations into the possible links between antimicrobial activity and photoluminescence [7]. To study the photocatalytic degradation of ZnO nanoparticles, methyl orange (MO) was used as a model pollutant. The decrease in the absorption maxima at 464 nm (Figure 7) indicates that ZnO nanoparticles are capable of photodegrading the organic dye. If the degradation ratio is defined as the ratio between the decreased absorptive intensity and the initial MO solution, the degradation ratio is about 91% for ZnO nanoparticles when the solution is irradiated for 150 min [42,45]. These results support the premise that daily exposure of aqueous suspensions containing organic pollutants and ZnO powder to the natural light could be efficient in degrading these pollutants.

**Figure 8:**
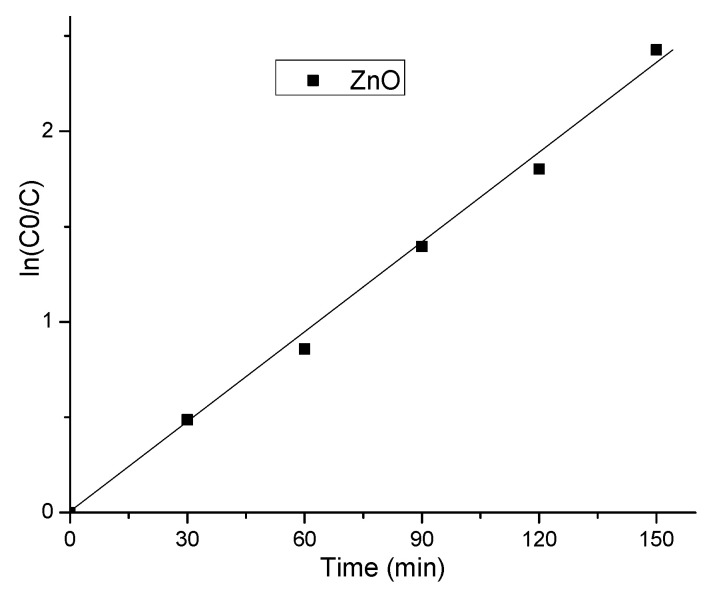
The pseudo-first-order rate constant *k*_app_ (min^−1^) was calculated from the slope of ln(C0/C) versus irradiation time *t*.

For diluted solutions, the photodegradation reactions exhibit apparent first-order kinetics (Figure 8),

ln(C0/C) = *k*_app_∙t (1)

where C0 is the initial concentration of MO, C is the MO concentration at time t (min), and *k*_app_ is the rate constant of the apparent first order.

The values for *k_app_*, removal efficiency, and the R-square of fitting data are given in Table 1.

The antibacterial mechanism of ZnO nanoparticles is still unclear, but it exhibits different activities in the presence of light than in the dark. In the presence of light, reactive oxygen species (ROS) are generated, and this might involve photocatalytic activity in the promotion of the antimicrobial activity. ROS are responsible for photocatalytic activity but also for oxidative stress, which is damaging to the bacterial membrane [46,47].

The antimicrobial activity of ZnO nanoparticles can be explained by the release of ROS from the surface of nanoparticles, photocatalytic activity, the release of Zn^2+^, direct contact with the cell membrane, and morphology or the size of the nanoparticles [3,32]. The release of ROS depends on the photocatalytic activity of ZnO nanoparticles because they are activated by UV and visible light. After the electrochemical reaction, ROS are generated [32,46,48]. Through the action of ROS at the level of the cell wall and membrane, the internalization of Zn^2+^ is facilitated, causing strong oxidative stress that determines the inhibition of cell growth, and finally leads to cell death [3,46,47,48].

The qualitative results obtained from evaluating the ZnO nanoparticles for antimicrobial activity revealed the appearance of the inhibition zone only in the case of the *S. aureus* (Table 2), with a diameter of the inhibition zone of 19 mm, which agrees with the literature [49]. The other two strains showed resistance to the action of tested samples, which can be explained by the greater sensitivity of Gram-positive bacteria vs. Gram-negative bacteria and yeast/fungi [3,47,50,51]. Gram-positive bacteria are more sensitive to the action of ZnO because of their simpler cell wall structure compared to other microorganisms, and the negative charge of the cell attracting positive ions (Zn^2+^) [3,47,52].

From the MICs evaluation of the tested suspensions, compared to the solid medium diffusion of the tested nanoparticles, the liquid medium diffusion enhances the level of manifestation of the inhibitory effect of the tested suspensions. The inhibitory effect for ZnO nanoparticles was displayed up to a concentration of 125 µg/mL in the case of *S. aureus*, which further manifested as the most sensitive strain to the action of this compound out of the three tested (Table 3) [53]. The MIC values for *S. aureus* were between 105 and 135 µg/mL and between 125 and 170 µg/mL for *E. coli* [54]. The MIC evaluation used the same types of standard bacterial strains as this study, obtaining a value of 500 µg/mL for both strains. Many studies show that Gram-negative bacteria and yeasts are more resistant to the action of ZnO nanoparticles [55,56,57,58].

**Figure 9:**
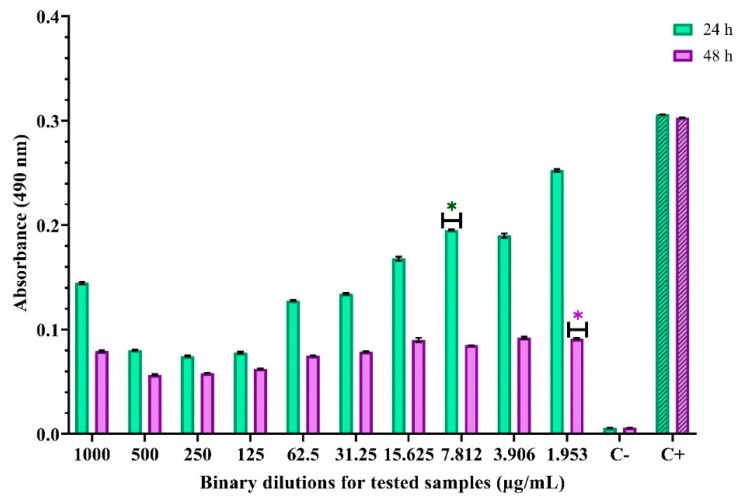
Absorbance values for evaluating anti-adhesion activity for ZnO nanoparticles against *S. aureus*. * indicates the MBEC values and differences between groups are considered statistically significant (*p* < 0.001).

We obtained quantitative results for the capacity to inhibit adhesion to an inert substrate. We analyzed the graphs obtained from the spectrophotometric reading of the absorbance at 490 nm to determine the minimal biofilm eradication concentration (MBEC) value. The inhibitory effect on the adhesion capacity to the inert substrate manifests differently depending on the strain. Thus, when the incubation was performed in the presence of different concentrations of ZnO nanoparticles, the adhesion of *S. aureus* to the inert substrate was intensely inhibited up to higher dilutions of the compound, with the correct MBEC being 7.812 µg/mL at 24 h and 1.953 µg/mL at 48 h (Figure 9).

**Figure 10:**
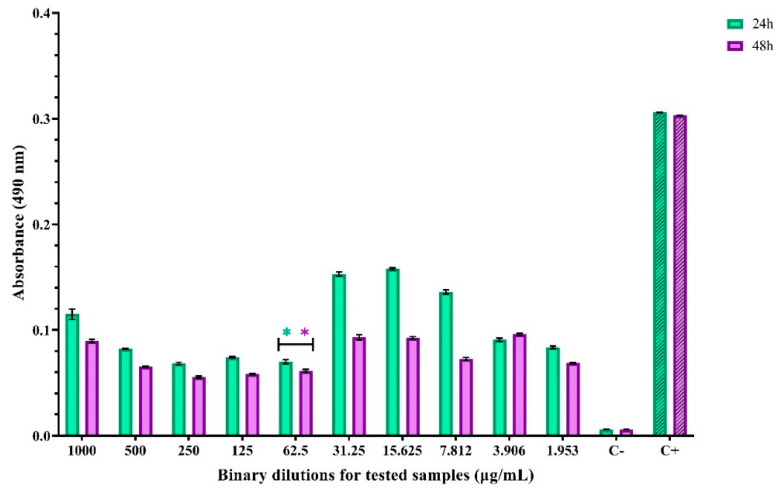
Absorbance values for evaluating anti-adhesion activity for ZnO nanoparticles against *E. coli*. * indicates the MBEC values and differences between groups are considered statistically significant (*p* < 0.001).

In the case of *E. coli*, the MBEC values at 24 h and 48 h after incubation were approximately equal (62.5 µg/mL), suggesting an impediment to the adhesion of *E. coli* cells to the inert substrate and, implicitly, to the development of a mature and stable biofilm (Figure 10).

**Figure 11:**
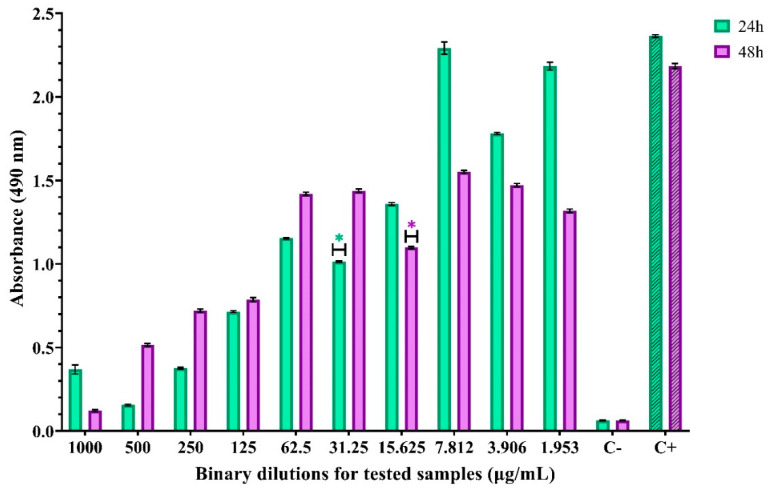
Absorbance values for evaluation of anti-adhesion activity for ZnO nanoparticles against *C. albicans*. * indicates the MBEC values and differences between groups are considered statistically significant (*p* < 0.001).

The inhibitory effect of ZnO nanoparticles against *C. albicans* was more pronounced than in previous assays and the MBEC value was 31.25 µg/mL at 24 h and 15.625 µg/mL at 48 h (Figure 11).

Biofilms are an accumulation of microbial cells that are surrounded by a matrix and attach to solid surfaces [59,60]. The first step in inhibiting biofilm formation is preventing surface colonization with antimicrobial agents. The antimicrobial potential of ZnO nanoparticles depends on the size of the nanoparticles because the smaller they are (<30 nm), the stronger the interaction with microbial cells, and the easier they penetrate the cell [60,61,62,63,64], which agrees with the XRD and TEM results obtained (20–30 nm diameter of nanoparticles). Another key finding of the antimicrobial effect of ZnO nanoparticles is the remarkable results obtained after the evaluation of the photocatalytic activity. The results revealed that Gram-positive bacteria (*S. aureus*) are highly sensitive to the action of ZnO nanoparticles compared to other strains and, therefore, they have antimicrobial potential in water treatment.

## 4. Conclusions

In conclusion, we presented zinc oxide nanoparticles that were successfully synthesized starting from zinc acetate dihydrate and sodium hydroxide, through a simple co-precipitation method. The X-ray diffraction pattern results prove that, based on the identified peaks, the material has a hexagonal wurtzite structure and exhibits good crystallinity. Furthermore, the FTIR analysis confirmed that the peaks from 462 and 419 cm^−1^ are attributed to the stretching vibrations of Zn–O, which indicate the presence of ZnO. From the electron microscopy images, it was observed that the ZnO nanoparticles tend to form topolyhedral-shaped agglomerates. It is worth mentioning that the size of the as-obtained zinc oxide nanoparticles was about 20–30 nm. For the evaluation of the photocatalytic activity of ZnO nanoparticles, methyl orange was used as a model pollutant, and it proved that ZnO has great photocatalytic activity against the organic dye. The antibacterial activity of ZnO nanoparticles was tested against Gram-negative and Gram-positive strains and it was found that the most activity was experienced against Gram-positive bacteria (*S. aureus*). ZnO nanoparticles obtained through the co-precipitation method have great potential as an antibacterial and photocatalytic agent for further use in developing nanocomposite membranes for water purification. It is expected that the absorptive property of these membranes will further contribute to the photocatalytic degradation of organic pollutants and the removal of antibiotics, pesticides, etc., found in wastewater that present a threat to both aquatic and human life.

## Figures and Tables

**Table 1:** The values for *k*_app_, removal efficiency, and R-square for ZnO nanoparticles.

Sample	*k*_app_ (min^−1^) 10^−3^	Removal Efficiency	R-Square
ZnO	15.82 ± 0.53	91%	0.995

**Table 2:** The diameters of the inhibition zones.

Bacterial Strains	The Diameter of the Inhibition Zone, mm
	ZnO NPs (10 mg/mL)
*Staphylococcus aureus* ATCC 25923	19
*Escherichia coli* ATCC 25922	0
*Candida albicans* ATCC 10231	0

**Table 3:** MIC values for the tested ZnO nanoparticles.

Bacterial Strains	ZnO NPs (10 µg/mL)
*Staphylococcus aureus* ATCC 25923	125 µg/mL
*Escherichia coli* ATCC 25922	250 µg/mL
*Candida albicans* ATCC 10231	500 µg/mL

## Data Availability

Not available.
